# In Vitro Effects of Short-Term and Long-Term Heat Exposures on the Immune Response and Prostaglandin Biosynthesis in Bovine Endometrial Cells

**DOI:** 10.3390/ani12182359

**Published:** 2022-09-09

**Authors:** Sroisuda Chotimanukul, Junpen Suwimonteerabutr, Mongkol Techakumphu, Theerawat Swangchan-Uthai

**Affiliations:** 1 Department of Obstetrics, Gynaecology and Reproduction, Faculty of Veterinary Science, Chulalongkorn University, Bangkok 10330, Thailand; 2 CU-Animal Fertility Research Unit, Chulalongkorn University, Bangkok 10330, Thailand

**Keywords:** endometrium, immune response, prostaglandin, cattle, heat exposure

## Abstract

**Simple Summary:**

The bovine endometrium is a major contributor to the regulation of reproduction and participates in several processes of producing offspring, including the resumption of estrus cyclicity, implantation and placenta formation. Heat stress is one of the underlying factors contributing to low fertility in cattle. The most significant elements of heat stress are increased temperature and humidity, which cause an economic loss in the dairy industry from decreased milk production, reduced reproductive performance and increased culling. The current study aimed to investigate the effect of elevated temperature (short-term or long-term) on bovine endometrial cell cultures. Our findings suggest that heat exposure compromised the endometrial immune response and prostaglandin synthesis in different ways based on elevated temperature duration, which could reduce subsequent fertility. This research provides data regarding the impact of thermal stress on uterine functions in cattle to further improve reproductive management strategies and prevent uterine infection in cattle experiencing heat stress during the current climate change era.

**Abstract:**

Worldwide heat stress (HS) conditions have a negative impact on dairy cow fertility. However, understanding of the effect of heat stress on endometrial functions is still unclear. The present study aimed to investigate the effects of differential heat exposure conditions on the immune response and prostaglandin biosynthesis of bovine endometrium challenged with bacterial lipopolysaccharide (LPS). Cultures of endometrial cells were grown to confluence at 37 °C (control) and 40.4 °C for 24 h after confluence (short-term heat exposure) and 40.4 °C for 8 days from the beginning of the culture (long-term heat exposure), prior to a challenge by 100 ng/mL LPS for 12 h. LPS altered *ALOX12*, *IL8*, *IL1B*, *S100A8*, *PTGES* and *AKR1B1* expressions, as well as secretory IL8 and PGF2α. Short-term heat exposure decreased *S100A8, IL8* and PGF2α compared with the control temperature, while long-term heat exposure decreased *S100A8* and PGF2α. In contrast, *HSPA5* expression was not altered by heat exposure or LPS. Indeed, the short-term heat treatment was insufficient for accomplishing the responses of the endometrium to LPS treatment for *IL8*, *S100A8* and *PTGES* expressions when compared with other temperature conditions. Our findings showed that heat exposure could compromise endometrium immune response and prostaglandin biosynthesis in different ways based on elevated temperature duration, which could reduce subsequent fertility.

## 1. Introduction

Declining fertility in dairy cattle during the past few decades has created enormous problems in the dairy industry worldwide. One of the main factors is the extreme genetic selection for milk production in dairy cattle, resulting in the era of high-yielding dairy cows that experience reproductive problems [1]. These problems are related to underlying causes, such as negative energy balance (NEB), postpartum endometritis and heat stress (HS) conditions. To date, the exact solution remains unclear, but there must be a multifactorial basis, including both genetic and environmental components.

Many lines of evidence reveal that HS is detrimental to bovine production and reproductive performance [2,3,4]. The major components that contribute to the level of HS in cattle are elevated temperature and humidity, denoted by the THI (temperature–humidity index) [5]. A previous study suggested that THI < 68 is generally optimal for healthy cows; THI of 68 to 74 causes mild discomfort conditions, and THI ≥ 75 is a severe HS indicator that reduced the fertility rate [6]. Selecting milk yield also reduces the thermoregulatory ability of cows during high ambient temperatures [7] and magnifies the seasonal detrimental effect on fertility caused by HS [8]. However, cattle across the world are affected by HS in different conditions: a long summer season in the southwestern United States and Brazil, and a short summer season or heatwave in central Europe, the northern United States and Canada [5,7]. Moreover, an association between HS and a decrease in the fertility of lactating dairy cows was found, particularly in the tropical areas with high ambient temperatures and humidity [9,10]. Although animals can adapt to HS, the response mechanisms, including cellular and molecular responses that ensure survival, are also detrimental to performance [11]. Thus, the adaptive responses to differential types of HS in particular cells may also act differently. Cows experiencing HS exhibit a sub-optimal uterine environment with a decreasing blood flow [12] and increasing uterine temperature [13], leading to failure of embryonic implantation. Additionally, because the development of oocytes and embryos is temperature-sensitive [14], the high temperature of the reproductive tract environment is likely to inhibit oocyte and embryo development [15,16]. Indeed, a recent study found that the group of cows that experienced HS (THI of ≥ 73) 3 weeks before insemination had more unfertilized oocytes than the control (21% vs. 4%, respectively), a lower conception rate, and reduced embryo development during the first 16 gestational days [17]. Moreover, HS can affect endometrial functions, e.g., prostaglandin and protein secretion [18,19].

After parturition, the endometrium undergoes extensive changes as a part of involution in order to prepare the uterus for the next pregnancy. At this time, the cow’s reproductive tract can easily be affected by local and metabolic factors, resulting in an apparent delay in the uterine clearance, repairing process, and subsequently affecting fertility [20]. Uterine clearance is one of the most important functions of the endometrium as bacterial contamination is a normal sequel to calving and is almost universal in dairy cows at the rate of 80–90% [21]. However, in postpartum cows, some parts of the endometrium are sloughed off. The adherence of microbes and colonization can then occur though a damaged surface epithelium, exposing the stroma to the pathogen. The bacteria responsible for endometritis are *Escherichia coli* (*E. coli*), *Trueperella pyogenes* and *Staphylococcus aureus* (*S. aureus*) [22,23].

Studies of bovine endocrine and immune response during uterine infection have been conducted because of the high incidence of uterine disease and its economic impact [24]. Cows with high uterine bacterial contamination also have high levels of circulating endotoxin lipopolysaccharide (LPS), which is absorbed following the accumulation of LPS within their uterine lumen [25]. The endometrium is defended against the microorganisms and their LPS toxins not only by physical barriers, but also by the production of several effectors, such as inflammatory mediators, antimicrobial peptides (AMPs), prostaglandin E2 (PGE2), prostaglandin F2alpha (PGF2α) and complement components [26,27,28,29]. In addition, LPS also enhances the production of oxidative stress (reactive oxygen species (ROS), nitric oxide (NO) and glutathione (GSH)) [30,31]. These molecules are capable of inducing postpartum uterine disorders [32], in particular mechanisms; for instance, PGE2 promotes the adhesion and invasion of *S. aureus* into endometrial cells [33], and oxidative stress promotes dysfunctional inflammatory responses [31,34]. Under a sub-optimal uterine environment caused by underlining causes, including HS, chronic endometritis may occur as a consequence of unresolved inflammation during involution [35]. This economically uterine disease compromised bovine fertility by causing sub-fertility, extending calving to conception intervals, increasing the number of services per pregnancy and increasing the herd culling rate [36].

We hypothesized that the HS in postpartum cows decreased the immune response, altered the resolution of inflammatory activity and disturbed the endocrine function of endometrial cells, resulting in poor fertility. We proposed that the LPS-treated primary cultures of bovine endometrial cells can be used to explore the effect of HS in terms of gene and protein expressions. Therefore, the present study aimed to investigate the effect of elevated temperature (short-term or long-term) conditions in LPS-treated bovine endometrial cell culture system on the expression of immune response genes and biosynthesis of prostaglandins. This research may provide us with findings on the impact of thermal stress on endometrial functions in dairy cattle to further improve reproductive management strategies and prevent uterine infection in cattle experiencing HS in the current climate change era.

## 2. Materials and Methods

All experimental procedures were conducted in accordance with the ethical standards issued by the National Research Council and approved by the Ethics Committee for Human and/or Animal Experimentation in meeting No.1531012, Faculty of Veterinary Science, Chulalongkorn University, Thailand.

### 2.1. Sample

Normal uteri from cross-bred Holstein Friesian cows (87.25–93.75% HF and Thai native cattle) in an early luteal phase were collected from non-pregnant cows at the slaughterhouse and then transported to the cell culture laboratory in a plastic bag with ice over the course of a few hours. The stage of the estrous cycle can be determined based on the appearance of the ovaries and genital tracts. The early luteal phase was characterized by the presence of corpus hemorrhagicum on the surface of the ovary [37]. The experiments were performed on four separate occasions.

### 2.2. Primary Bovine Endometrial Cells Culture

Primary endometrial epithelial and stromal cells were isolated from the endometrium and then cultured using previously described methods [29]. Briefly, the external parts of the uteri were washed in 70% ethanol after arriving at the laboratory. Then, the endometrial tissues were separated before being cut into strips. The endometrial tissues were chopped into 1 mm^3^ pieces with a surgical blade and transferred to Hanks’ balanced salt solution (HBSS, Gibco, Thermo Fisher Scientific, Waltham, MA, USA). After pooling in a sterile beaker, the tissues from each uterus were then digested into a cell suspension by a 150 mL sterile digestive solution, made by dissolving 50 mg Trypsin (Worthington Biochemical Corporation, Lakewood, NJ, USA), 50 mg Collagenase A (Roche), 100 mg bovine serum albumin (BSA; Sigma-Aldrich Company Ltd, Dorset, UK) and 10 mg Deoxyribonuclease (DNase) I (Roche) into 100 mL HBSS. After incubation for 90 min at 37 °C in a 5% CO_2_ incubator (shaking every 10 min in between), the cell suspension was filtered through a 70 µm cell strainer (Falcon, Fisher Scientific). The filtered cell suspension was re-suspended with HBSS containing 10% fetal bovine serum (FBS; Sigma) and centrifuged at 100 g for 10 min prior to repeating the washing procedure twice. After checking the cell integrity and number by trypan blue staining, the washed pellet was re-suspended with Dulbecco’s modified Eagle’s medium (DMEM/F12; Gibco, Thermo Fisher Scientific), a basic medium prepared by adding 50,000 U of penicillin and streptomycin to 500 mL DMEM/F-12 containing 10% FBS. After pooling the cell suspension, 1 mL of cell suspension at 5 × 10^5^ cells was allocated into each well of 24-well plates (Nunc, Thermo Scientific) and another 1 mL of DMEM/F12 medium containing 10% FBS was added to each well. The culture media were changed every 48 h until the cells reached confluence.

### 2.3. Characterization of Endometrial Culture System

**Figure 1:**
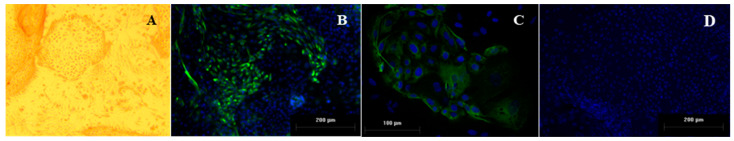
Phase contrast and immunofluorescence photomicrographs of bovine mixed endometrial and stromal cells. (**A**) Epithelial colony surrounded by stromal cells. (**B**) With anti-vimentin antibody, stromal cells were specifically stained with fluorescent (green) with Hoechst labeling of the nuclei (blue). (**C**) With anti-cytokeratin antibody, epithelial cells were specifically stained with fluorescent (green). (**D**) Representative images of negative control of primary antibody used in this study.

Primary endometrial cell cultures were previously validated using immunocytochemical staining to identify specific cell types [29]. Endometrial stromal cells were stained by anti-vimentin-clone V9 (Dako), while the epithelial cells were not stained with anti-vimentin; epithelial cells were stained by anti-human cytokeratin-clone AE1/AE3 (Dako), whereas immune cells were stained positive by anti-CD172a (DH59B; Monoclonal Antibody Center VM&P, Washington State University, Pullman, WA, USA). The relative proportions of each cell type after 6 days of culture were evaluated using image analysis software (ImageJ version 1.44; Research Services Branch, NIMH/NIH, Bethesda, MD, USA). The cultures with an epithelial to stromal cell ratio of approximately 59:41 on day 6 of culture when the heat exposure experiments were performed, in addition to a negligible presence of contaminant immune cells (<0.00001%), were used for further study. Due to the location change of the cell culture laboratory, the cell cultures were re-validated. The presence of cultured endometrial epithelial and stromal cells in this experiment is illustrated in Figure 1. In a monolayer, 2 types of endometrial cells can be visualized by phase contrast microscopy (Figure 1A). Stromal cells may overgrow each other in multiple layers, and epithelial cells usually aggregate in large clumps. Epithelial cells cultured on plastic typically appear cuboidal in shape, as seen in the center of Figure 1A. The patch of epithelial cells was surrounded with spindle and fibroblast-shaped cells that were of stromal origin. Using immunofluorescence staining for vimentin, the stromal cells were strongly positive (Figure 1B), while the epithelial cells were positively stained with anti-cytokeratin (Figure 1C). The nuclei of all cells were stained blue with Hoechst (Figure 1B–D). Accordingly, day 7 of cultured cells of our culture system showed a suitable proportion of mixed stromal and epithelial cells in this system (~1:1); therefore, they were used in the current experiment.

### 2.4. Cell Culture Treatment with Heat Exposure and LPS

The experiment focuses on the in vitro effects of the differential heat exposure conditions on bovine endothelial cells challenged with LPS (from Escherichia coli O55:B5, Sigma Chemical Inc., St Louis, MO, USA, L2880). The treatment with heat exposure was classified into short-term and long-term treatment. On day 0, cells were isolated, suspended in DMEM/F12 basic medium with 10% FC serum, and seeded in three 24-well plates (2 plates were incubated at 37 °C and 1 plate at 40.4 °C (long-term heat exposure). The medium was replaced after 48 h and then every 2 days until the cells reached confluence in 7 days. One plate from the 37 °C incubator was moved to 40.4 °C for 24 h (short-term heat exposure). Then, the media of all plates were replaced (1.5 mL/well) with DMEM/F12 basic medium in the presence or absence (control) of LPS (Sigma) 100 ng/mL. An aliquot and cells were taken 12 h after LPS challenging. Treatments were replicated three times, and experiments were conducted on four separate occasions. The results and statistical analysis were performed to compare the effects of LPS and elevated temperature based on 12 replications in each treatment/control group.

### 2.5. Optimization of Endometrial Cell Culture and Exposure to Elevated Temperature

**Figure 2:**
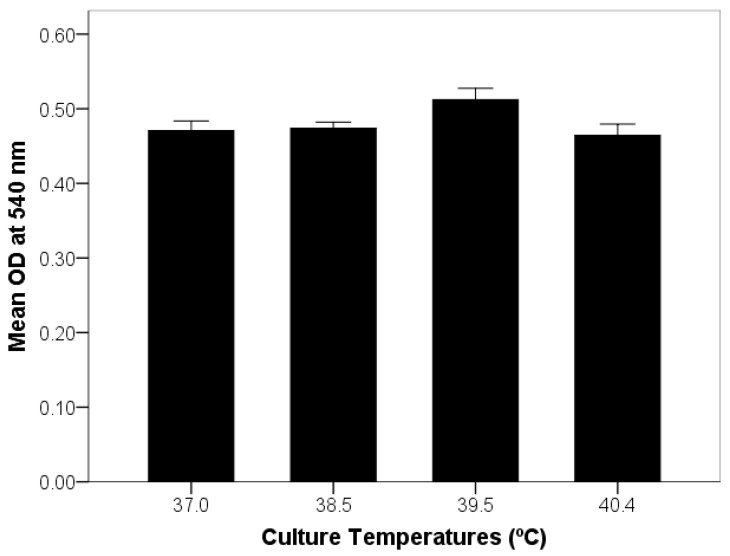
Cytotoxicity effect of the culture temperature on the viable cells of mixed endometrial cell culture. OD: optical density readings at 540 nm. Values are mean ± SEM.

Prior to the start of the actual endometrial cell culture experiment, a preliminary study of mixed stromal and epithelial cell culture was conducted to determine the number of viable cells affected by heat exposure. Cultures of mixed epithelial and stromal cells were grown to confluence in 96-well plates with the procedure described above. Tests of heat exposure were performed by exposing the set of 6 culture wells to different temperature conditions of 37 (control temperature), 38.5, 39.5 and 40.4 °C for 24 h. The relative number of viable cells was determined using the CellTiter 96 AQueous One Solution Cell Proliferation Assay (Promega, Southampton, UK). The absorbance (optical density, OD) of samples and standards was read at 540 nm on a microtiter plate reader (Spectra MAX 250 Molecular Devices, Sunnyvale, CA, USA). Data showed that all elevated temperature conditions did not affect the number of viable cells in culture compared to the control temperature (Figure 2), and thus the temperature at 40.4 °C was used for the treatment group in the current study.

### 2.6. Sample Collecting for Evaluation

Mixed stromal and epithelial cells from each plate were collected in order to evaluate mRNA expression by qRT-PCR. The supernatant from each well was collected separately, aliquoted into two 1.5 mL Eppendorf tubes, and stored at −20 °C before measuring IL8 and PGF2α using an enzyme-linked immunosorbent assay (ELISA).

### 2.7. Quantitative Real-Time PCR System

The procedures of RNA isolation, reverse transcription (RT) and qPCR were performed as previously described [29,38].

#### 2.7.1. RNA Isolation and Reverse Transcription

Total RNA was extracted from the cell culture using the column method, with RNeasy Mini Kits (QIAGEN) in accordance with guidelines supplied by the manufacturer as previously described [29]. The concentration and purity of RNA samples were determined using an ND-1000 NanoDrop spectrophotometer (NanoDrop Technologies, Wilmington, DE, USA), while RNA integrity was confirmed by agarose gel electrophoresis. In the following stage, 1000 ng of total RNA from each sample was stored at −80 °C until it was treated for genomic DNA carryover with an RNase-free DNase kit (Promega) according to guidelines supplied by the manufacturer. Subsequently, the DNase-treated RNA was reverse transcribed into cDNA using random hexamers (Reverse Transcription System kit; Promega).

#### 2.7.2. Primer Sequences and Optimization of qPCR

Oligonucleotide primer sequences were obtained from previous references [29,39] or newly designed (Table 1). For the optimization of qPCR, primers were tested by conventional PCR amplification using GoTaq^®^ Green Master Mix (Promega). The presence of a single band of DNA by electrophoresis on a 1% (*w*/*v*) agarose gel confirmed the specificity of PCR products without primer dimers.

#### 2.7.3. Real-Time qPCR

The expressions of seven candidate genes (*ALOX12, HSPA5*, *IL8*, *IL1B*, *S100A8*, *PTGES* and *AKR1B1)* and reference genes (*RN18S1* and *ACTB*) were determined by the absolute real-time qPCR method (CFX96 Real-Time PCR Detection System, Bio-Rad Laboratories, Inc., Hercules, CA, USA). The master-mix (KAPA SYBR^®^ FAST qPCR kit: KAPA Biosystems) was prepared once for each assay in order to avoid errors from pipetting. Standards for qPCR were prepared from purified PCR products using a QIAquick PCR purification kit (Qiagen). The PCR reactions were run in duplicate, and data were evaluated in a closed white tube in a 96-well plate. A no-template control (NTC) using nuclease-free water was included on every plate. The program of thermal cycling conditions applied to each assay consisted of an initial Taq activation step at 95 °C for 15 min followed by 38 cycles of denaturation, annealing, extension and plate reading. The absolute copy number of the PCR product was achieved by comparing the CT values of the unknown samples to a standard curve using the Bio-Rad CFX Manager software version 3.1. The gene expression of each target gene was determined using the GeNorm normalization algorithm against two selected reference genes.

### 2.8. Measurement of Secreted Protein Using ELISA for IL8 and PGF2α

Samples of supernatant of the cell culture system were used to determine IL8 levels in duplicate using Human CXCL8/IL-8 DuoSet ELISA (R&D Systems, Inc., Minneapolis, MN, USA) in accordance with the guidelines supplied by the manufacturer. This IL8 kit is proven to effectively measure bovine IL8 with an acceptable cross-reactivity. All samples were measured on the same occasion. The intra-assay coefficient of variation (CV) was 5.08%, and the detection limit was 6.44 pg/mL. The PGF2α measurement was determined in duplicate by using the Prostaglandin F2 alpha ELISA Kit (Abnova) in conformity with the guidelines supplied by the manufacturer. The intra-assay coefficient of variation (CV) was 8.26%, and the detection limit was 6.71 pg/mL.

### 2.9. Statistical Analysis

The amount of mRNA expression, IL8 and prostaglandin level were analyzed using IBM SPSS Statistic Version 22.0 (SPSS; IBM, Armonk, NY, USA). Data were tested for homogeneity of variance using a Levene’s test, and log transformation was performed if necessary. ANOVA with a randomized block design via a linear mixed model analysis was used to evaluate 2 fixed effects—LPS and the elevated temperature conditions—followed by Bonferroni post hoc pairwise comparisons. The batch of cell cultures was included as a random effect. Data are presented as sample mean ± standard error (SEM) and plotted as bar charts. Significance levels were set as the cut-off for *p*-values at 0.05. Data are illustrated with GraphPad Prism 9.2.0 (GraphPad Software, San Diego, CA, USA).

## 3. Results

### 3.1. Effect of Heat Exposure and LPS on Gene Response of Bovine Endometrial Cells

Concentrations of *ALOX12*, *HSPA5*, *IL8*, *IL1B*, *S100A8*, *PTGES* and *AKR1B1* were measured in all samples as absolute values using reverse transcriptase qPCR. In addition, *ACTB* and *RN18S1* were included as reference genes. All treatments did not alter *ACTB* and *RN18S1* mRNA expressions. Thereby, the data for the mRNA expressions of the measure genes were normalized with a normalization factor of *ACTB* and *RN18S1* using geNorm [40]. Gene and protein expression data of the bovine mixed epithelial and stromal cells with the presence of LPS and heat exposure conditions (short-term/long-term) are shown in Table 2. LPS significantly altered the mRNA expressions of *ALOX12*, *IL8*, *IL1B*, *S100A8*, *PTGES* and *AKR1B1* (*p* < 0.01) in endometrial cells across all combined culture temperature conditions. In regard to temperature conditions, short-term heat exposure decreased *S100A8* expression and secretory IL8 and PGF2α (*p* < 0.05) compared with the control temperature group, while long-term heat exposure decreased *S100A8* expression and secretory PGF2α (*p* < 0.05) compared with control temperature group.

Interestingly, short-term heat exposure was insufficient for significantly increasing the expressions of *IL8*, *S100A8* and *PTGES* (*p* > 0.05) in cultures treated with LPS when compared with control and long-term heat exposure conditions. In contrast with the short-term heat exposure group, LPS increased *ALOX12*, *IL1B*, *S100A8* and *PTGES* expressions, while significantly decreasing the expression of *AKR1B1* (*p* < 0.01) in the long-term heat exposure group. Regarding data focusing on the endometrial cells in the absence of LPS, the long-term heat treatment significantly decreased *S100A8* expression (*p* < 0.05), but increased *AKR1B1* expression (*p* < 0.05). Neither heat exposure nor LPS, however, had any effect on *HSPA5*.

### 3.2. Effect of Heat Exposure and LPS on Secretory IL8 of Bovine Endometrial Cells

**Figure 3:**
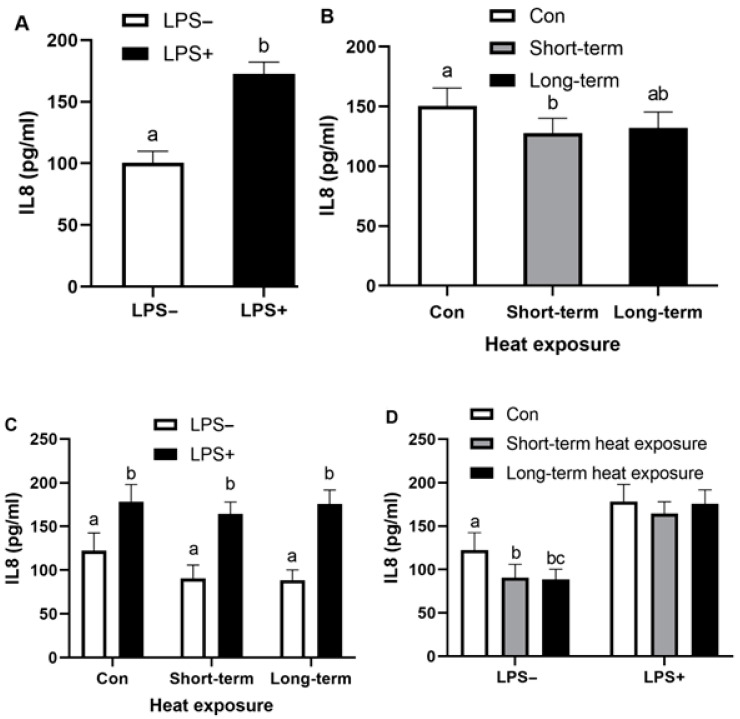
Secretion of IL8 (pg/mL) in the medium of bovine endometrial cells cultured in the presence or absence of 100 ng/mL LPS with different heat exposure (control: Con; short-term heat exposure; long-term heat exposure). Treatments were replicated three times, and the experiment was conducted on four separate occasions. Different superscripts indicate significant differences *(p <* 0.05), as determined by mixed model analysis using two fixed factors—LPS treatment (**A**) and heat exposure (**B**)—followed by Bonferroni post hoc test (**C**,**D**). Values are mean ± SEM.

Data of secretory IL8 in the culture supernatant of mixed endometrial epithelial and stromal cells after LPS treatment in different conditions of heat exposure condition (short-term and long-term) are shown in Figure 3 and Table 2. LPS significantly up-regulated the secretion of IL8 (*p* < 0.01), particularly in each temperature condition (Figure 3A,C). Regarding the effect of heat exposure, only short-term heat exposure decreased the IL8 level *(p <* 0.01; Figure 3B). Indeed, both short-term and long-term heat exposures reduced the IL8 level *(p <* 0.05) in the non-LPS-treated endometrial cells (Figure 3D).

### 3.3. Effect of Heat Exposure and LPS on Secretory PGF2α of Bovine Endometrial Cells

**Figure 4:**
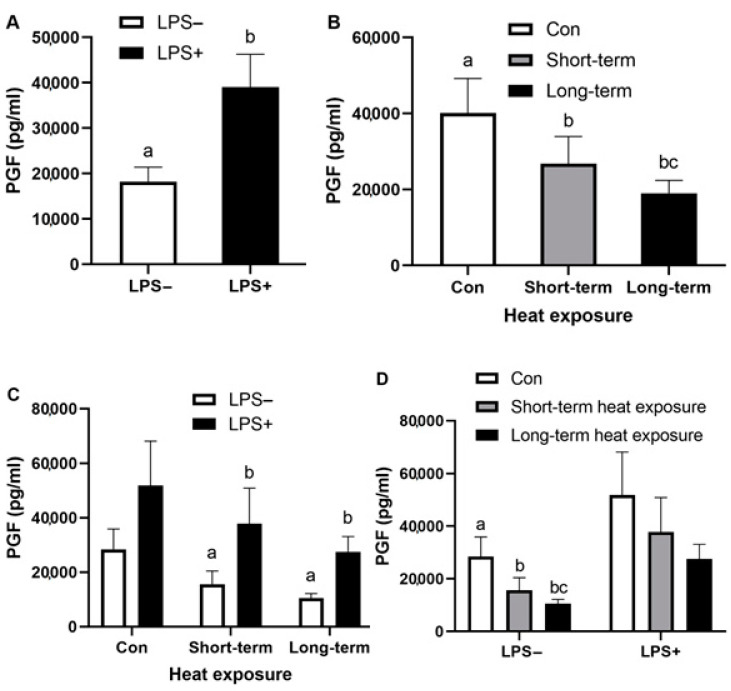
Secretion of PGF2α (pg/mL) in the medium of bovine endometrial cells cultured in the presence or absence of 100 ng/mL LPS with different heat exposure (control: Con; short-term heat exposure; long-term heat exposure). Treatments were replicated three times, and the experiment was conducted on four separate occasions. Different superscripts indicate significant differences *(p <* 0.05), as determined by a mixed model analysis using two fixed factors—LPS treatment (**A**) and heat exposure (**B**)—followed by Bonferroni post hoc test (**C**,**D**). Values are mean ± SEM.

PGF2α concentrations were detected by ELISA in the culture supernatant of mixed endometrial cells after LPS treatment in different conditions of heat exposure. Data comparing the PGF2α level of endometrial cells treated with LPS and heat exposure conditions (short-term and long-term) are shown in Figure 4. LPS significantly up-regulated the secretion of PGF2α, especially in the cells cultured with elevated temperature conditions (*p <* 0.01; Figure 4A,C), while both heat exposure conditions decreased PGF2α concentrations *(p <* 0.05; Figure 4B). Regarding the non-LPS treated group, both elevated temperature conditions decreased the secretion of PGF2α in endometrial cell cultures *(p <* 0.05; Figure 4D).

## 4. Discussion

Normal uterine functions are crucial for the successful production of new offspring. Several factors that initiate a sub-optimal uterine environment could cause unfertilization, embryonic loss and a low conception rate [41]. Endometrial tissue is mainly composed of epithelial and stromal cells that have a distinct morphology and show functional differences in response to several inflammatory mediators [42]. In the present study, the culture system of LPS challenging endometrial cells was used as a reference for normal endometrial function in response to bacterial endotoxin. As expected, LPS significantly modulated the mRNA expression of pro-inflammatory cytokine *IL1B*, chemokine *IL8*, antimicrobial peptide (AMP) *S100A8*, pro-resolution molecule *ALOX12* and PG synthases *PTGES* and *AKR1B1,* as well as the secretory IL8 and PGF2α concentrations in the cultures of bovine mixed endometrial epithelial and stromal cells. In accordance with the previous studies [29,39,43,44], *IL1B, IL8* and *S100A8* mRNA expressions were markedly increased in bovine endometrial cells cultured with LPS.

The underlying causes of sub-fertility have been explored for many years. An association between HS and a decline in the fertility of lactating dairy cows was found, particularly in tropical areas with high ambient temperatures and humidity [8,45]. An in vitro model of bovine endometrium in the current study was developed to investigate the effects of thermal stress on the innate immune response and prostaglandin biosynthesis. The body temperature of dairy cattle is approximately 38.5 °C, but increases under high ambient temperatures [46]. The critical temperature for the beginning of heat stress in high-yielding dairy cows in a sub-tropical climate is between 25 and 26 °C [7], combined with the effects of humidity, which further decrease performance. A previous study [10] reported that uncooled cows raised in Thailand had a high vaginal temperature during the day, at an average of 39.5 °C (ranging from 39.0 to 40.3 °C), which returned to a normal vaginal temperature (below 39.0 °C) late at night and during the early morning. Temperatures up to 40.4 °C did not significantly alter the number of viable cells in our mixed endometrial cells culture system, and thus this value was used as the heat exposure temperature in the current study. In addition, the time point of 12 h for LPS-challenged endometrial cultures was selected based on a previous study [29], where most of the immune-related genes and prostaglandin synthase genes expressed significantly different mRNA levels at 12 h and then decreased by 48 h.

HS can be classified as a short-term or long-term condition that affects all physiological functions and increases the expression levels of inflammatory cytokines and heat shock proteins (HSPs) [47,48]. To the best of our knowledge, this study provides the first evidence that short-term heat exposure was insufficient for significantly increasing the expression of *IL8*, *S100A8* and *PTGES* in cultures treated with LPS when compared with control and long-term heat exposure conditions. Nevertheless, LPS significantly up-regulated the secretion of IL8 in each temperature condition. In contrast, long-term heat exposure seems to have a strong negative impact on the group of non-LPS treated endometrial cells, decreasing the expressions of *S100A8* and secretory IL8. IL8 is a potent chemotactic factor recruiting polymorphonuclear cells (PMNs) to the site of inflammation [49,50]. The expression levels of *IL8* and *S100A8* are shown to be important markers that reflect the infection status of a cow when inflammation is established [20,51]. To eliminate uterine bacterial infection, immune cells need to be recruited to an epithelial layer [52], and neutrophils play a pivotal role in the elimination of these bacteria by the chemoattractive effect of IL8 [53,54] and the antimicrobial peptide *S100A8* [29]. In addition, the deletion of the *S100A8* gene in mice results in embryo resorption [55]. As a result, data from the present study suggest that thermal stress could directly enhance endometrial defense mechanisms and fetal–maternal interactions, which subsequently resulted in reduced fertility. A previous study also found that the HS caused an alteration of the chemokine production in bovine endometrial epithelial and stromal cells with the different mechanisms. HS altered IL8 in endometrial stromal cells, but not in endometrial epithelial cells [54]. However, the presence of mixed epithelial and stromal cells in the culture system of the current study would have enabled interactions between the two cell types to occur, resembling the in vivo situation.

Endometrial PG production initiates a normal luteolytic mechanism and estrus cyclicity. Increased PGF2α and metabolite PGFM concentrations were found in cows experiencing a postpartum uterine infection. Moreover, a recent study showed that HS created a sub-optimal uterine environment, which leads to increased PGFM, increased unfertilized eggs and reduced embryo development [5]. In the physiological activity of endometrial cells, HS-modulated endometrial prostaglandin and protein secretion selectively increased PGF2α synthesis rather than PGE2 and reduced conceptus protein synthesis [56]. A recent study with only short-term heat exposure [57] reported that an elevated temperature (39.5 or 40.5 °C) for 10 h doubled the gene expressions of enzymes involved in prostaglandin synthesis, including *PLA2*, *COX2*, *PGFS*, *PGES* and *CBR1*, resulting in an increase in the PGE2 and PGF2α production of endometrial stromal cells, except in endometrial epithelial cells. In contrast with the present study, we found that mixed endometrial epithelial and stromal cells responded to both heat exposure conditions by decreasing secretory PGF2α, particularly in the cultures without LPS, whilst LPS up-regulated the secretion of PGF2α, especially in the cultures with elevated temperature conditions. Indeed, the data show that the expression of PGE synthase *PTGES* was not fully up-regulated by an acute heat exposure in LPS treated-endometrial cells, whereas long-term heat exposure significantly decreased PGF synthase *AKR1B1* expression. Though there were some differences between the current study and previous studies in the data from the prostaglandin biosynthesis pathway, which may be due to variations in the experimental design, the main finding among these studies was a raised baseline of PGF2α secretion without pulsatile pattern, contributing to the inhibition of normal luteolysis [58,59].

The role of *ALOX12* (also known as *12-LOX*) has been implicated in various inflammation-related diseases. It is interesting to note that the LPS-induced expression of pro-inflammatory cytokines *IL6*, *IL12*, *CXCL9* and *CXCL10* was reduced by the inhibition of *ALOX12* in macrophages [60,61]. Data from the present study show that *ALOX12* was up-regulated in mixed endometrial cell cultures challenged by LPS for 12 h, but the thermal stress had no impact on the expression of this molecule. The reason for this finding is that there are controversial reports of *ALOX12* regarding inflammation, as its metabolites demonstrate both pro- and anti-inflammatory activities. Mechanisms by which *ALOX12* exhibits its pro-resolving effects are not fully understood. The direct effect of LPS on *ALOX12* expression was evident here, but HS may affect other pro-resolution molecules, e.g., lipoxins, resolvins and protectins, which exert potent and direct anti-inflammatory effects in various cell types [61,62,63,64]; therefore, they should be further investigated.

Due to their protective role against heat stress-induced cell damage, HSPs in cattle have been studied by numerous research groups in recent decades. HSPs are therefore used as heat stress markers [65]. In this study, neither heat exposure nor LPS had any impact on *HSPA5* (heat shock protein family A (Hsp70) member 5), whilst previous studies showed that the expressions of heat stress markers *HSP70* and *HSP90* were increased in bovine endometrial explants cultured in vitro under heat stress conditions [56,66]. Another in vivo study also found that endometrial *HSP70* was higher in the summer than in the winter of the Southern Japan [57]. The reason for this difference is still unclear. We based our results on the endometrium retrieved from dairy cattle in Thailand that have been raised and genetically adapted to tropical conditions over multiple generations. Moreover, the genotype and allele frequency of HSP70 polymorphisms [67] revealed that genotype AA is more frequent in Thai Friesian dairy cows than genotype AB; therefore, these cows are more tolerant to heat stress.

Based on our in vitro findings and previous in vivo studies, the suggested strategies that attempt to decrease the production loss and other effects of HS in high-yielding dairy cattle should depend on the type of heat exposure. For the short-term effect of heat exposure conditions, e.g., short summer seasons or heatwaves in some regions, the cows’ gene expressions related to uterine defense mechanisms were dramatically reduced, which may result in postpartum uterine infection. In addition, high-production cows in early lactation are sensitive to HS, and their production remarkably declined when their body temperature exceeded 39 °C for more than 16 h [68]. Consequently, environment management by adjusting cooling management systems on farms and nutritional dietary manipulation might be a valid option [69,70]. Long-term heat exposure occurs when dairy cows are raised in tropical and sub-tropical regions. Our results show that the immune-related gene and protein expressions in the endometria of cows were altered. The climatic conditions of the tropics bring about an increase in temperature and relative humidity. In combination with the high production of metabolic heat from body maintenance and milk production, postpartum cows will suffer from an extreme heat load, resulting in a decline in feed and water intake, a high body temperature and a decrease in fertility [9]. The strategic management of this condition should focus on the use of an appropriate genetic approach [71] by improving reproductive traits without disturbing production in high-producing dairy cattle under heat stress conditions.

## 5. Conclusions

In summary, a better understanding of the molecular mechanisms of bovine reproduction and its alteration by HS, especially in the local uterine function, is needed to improve the strategies of reproductive management and develop an innovative approach regarding dairy herd health to prevent a sub-optimal uterine environment associated with low fertility rates. To our knowledge, the current study provides the first evidence that bovine endometrial cells responded to bacterial endotoxin in different ways based on an elevated temperature duration under in vitro conditions. Short-term heat exposure decreased antimicrobial peptide *S100A8* gene expression and secretory IL8 and PGF2α compared with the control temperature, while long-term heat exposure reduced *S100A8* expression and secretory PGF2α. Notably, the short-term heat treatment was insufficient for fully accomplishing the responses of bovine endometrial cells to LPS treatment in terms of *IL8*, *S100A8* and prostaglandin E synthase (*PTGES*) expressions when compared with control and long-term heat exposure conditions.

## Figures and Tables

**Table 1:** Oligonucleotide primer sequences, GenBank accession, expected amplicon size, specific annealing temperature, plate read temperature, R^2^ of the standard curve and efficiency of the assay (E) used for RT-PCR determination. All primers were finally accessed in May 2022.

Gene #.	Primer Sequence (5′ → 3′)	GenBank Accession	Product Length (bp)	Annealing Temp (°C)	R^2^	E (%)
*ALOX12*	F: TCTCTACGCCTGTGATGCTTTA	NM_001192336.1	202	55	0.986	99.63
	R: GAGTTGATTCTGGGACTGGAAG					
*HSPA5*	F: GGTATTGAAACTGTGGGAGGTG	NM_001075148	162	62.5	0.924	100.2
	R: AAGGTGATTGTCTTTCGTCAGG					
*IL1B*	F: CTTGGGTATCAAGGACAAGAAT	NM_174093.1	207	62.5	0.991	99.7
	R: TCGATTTGAGAAGTGCTGATGT					
*IL8*	F: CCTAGTCTTGCCCTTATTATGC	NM_173925.2	232	62.5	0.998	101.6
	R:ACCAGTAGAAAGAACTGTGAACAT					
*S100A8*	F: TGCCATTAACTCCCTGATTGAC	NM_001113725.1	179	53	0.997	98.35
	R: TAATTCCACCATCCTGATTGAT					
*PTGES*	F: TGGTCATCAAAATGTACGTGGT	NM_174443.2	201	59.5	0.976	99.9
	R: AGTAGACAAAGCCCAGGAACAG					
*AKR1B1*	F: GTCTCCAACTTCAACCATCTCC	NM_001012519.1	250	59.5	0.979	102.1
	R: TGTACTTGTCTGCAATCGCTTT					
*RN18S1*	F: CGGCGACGACCCATTCGAAC	NR_036642.1	99	51	0.959	99.9
	R: GAATCGAACCCTGATTCCCCGTC					
*ACTB*	F: CATCGCGGACAGGATGCAGAAA	NM_173979.3	158	59.5	0.946	98.2
	R: CCTGCTTGCTGATCCACATCTGCT					

# Gene products analyzed were arachidonate 12-lipoxygenase, 12S type (*ALOX12*); heat shock 70 kDa protein 5 (*HSPA5*); interleukin 8 (*IL8* or *CXCL8*); interleukin 1, beta (*IL1B*); S100 calcium-binding protein A8 or calgranulin-A (*S100A8*); prostaglandin E synthase (*PTGES*); aldo-keto reductase family 1, member B1 (*AKR1B1*); *RN18S1* (18S ribosomal RNA); and actin, beta (*ACTB*).

**Table 2:** Comparison of mRNA expression (expressed in fg/µg reverse-transcribed RNA) and concentrations of secretory IL8 and PGF2α (pg/mL) on the bovine endometrial cells regarding the effects of heat exposure conditions in the presence of LPS (100 ng/mL for 12 h) ^#^.

*Gene* Symbol/	LPS	Expression	Heat Exposure (mRNA Expression)		
Protein			Control	Short-Term	Long-Term
*ALOX12*	−	27094 ± 3449	28675 ± 5812	28693 ± 7056	23913 ± 5345
	+	51313 ± 7287 *	45365 ± 10268 *	56026 ± 13745 *	52549 ± 14390 *
		Combined effect +	37020 ± 6026	42360 ± 8075	38231 ± 8079
*HSPA5*	−	1142994 ± 97170	1333116 ± 211903	973895 ± 156725	1121972 ± 121398
	+	1393799 ± 102820	1516190 ± 236571	1374429 ± 134236	1290779 ± 156742
		Combined effect +	1424653 ± 156477	1174162 ± 109208	1206375 ± 98534
*IL1B*	−	3416 ± 919	4138 ± 2478	3283 ± 953	2773 ± 763
	+	9853 ± 2915 *	10559 ± 4430	8658 ± 3296	10341 ± 7100 *
		Combined effect +	7349 ± 2571	5970 ± 1769	6721 ± 1582
*IL8*	−	84364 ± 26922	76294 ± 27655	61850 ± 16574	114946 ± 75776
	+	189605 ± 38673 *	216197 ± 75931 *	116970 ± 27697	235649 ± 83705 *
		Combined effect +	146246 ± 42123	89410 ± 16798	175297 ± 56630
*S100A8*	−	8204 ± 1441	11769 ± 3163 ^a^	9595 ± 2383 ^a^	3248 ± 757 ^b^
	+	22952 ± 4626 *	31561 ± 11748 *	15243 ± 3582	22053 ± 6418 *
		Combined effect +	21665 ± 6297 ^a^	12419 ± 2185 ^b^	12651 ± 3719 ^b^
*PTGES*	−	34703 ± 3097	28112 ± 4580	37523 ± 5786	38473 ± 5582
	+	46914 ± 4294 *	43547 ± 10126 *	51730 ± 7728	45464 ± 3227 *
		Combined effect +	35829 ± 5668	44626 ± 4948	41969 ± 3236
*AKR1B1*	−	40722 ± 3621	31479 ± 1946 ^a^	40621 ± 6400 ^a,b^	50067 ± 8044 ^b^
	+	31284 ± 1895 *	28818 ± 3224	33273 ± 3596	31759 ± 3168 *
		Combined effect +	30148 ± 1862	36947 ± 3671	40913 ± 4639
IL8	−	100.6 ± 9.4	122.4 ± 20.2 ^a^	90.7 ± 15.2 ^b^	88.6 ± 11.6 ^b^
(pg/mL)	+	172.8 ± 9.4 *	178.3 ± 19.7 *	164.4 ± 13.5 *	175.7 ± 16.1 *
		Combined effect +	150.4 ± 15.0 ^a^	127.5 ± 12.6 ^b^	132.1 ± 13.3 ^a,b^
PGF2α	−	18224 ± 3174	28436 ± 7454 ^a^	15663 ± 4768 ^b^	10574 ± 1578 ^b^
(pg/mL)	+	39060 ± 7193 *	51854 ± 16261	37856 ± 13075 *	27473 ± 5597 *
		Combined effect +	40145 ± 9082 ^a^	26759 ± 7188 ^b^	19024 ± 3345 ^b^

# Values are mean ± SEM of 12 samples from three experimental replicates on four separate occasions per treatment measured in duplicate by qPCR. Data were analyzed with a randomized block design via linear mixed models followed by Bonferroni post hoc tests between the effect of heat exposure conditions and LPS treatment. + (within-row) shows the combined effect of LPS treatment across all culture temperature conditions combined; (within-column) shows the combined effect of heat exposure across all LPS treatments combined. * Significant differences (*p* < 0.01) of LPS compared to the control condition. ^a,b^ Significant differences (*p* < 0.05) between heat exposure conditions are expressed with different superscript letters.

## Data Availability

Not applicable.
